# Supplementary material for: Bovine Parainfluenza Virus Type 3 (BPIV3) Enters HeLa Cells via Clathrin-Mediated Endocytosis in a Cholesterol- and Dynamin-Dependent Manner
Source: Viruses. 2021 May 31;13(6):1035. doi: 10.3390/v13061035 (PMC8228847; doi:10.3390/v13061035)
Supplement: Supplementary file 1 [file viruses-13-01035-s001.zip › viruses-1201262-supplementary.pdf]

viruses

Supplementary Figures

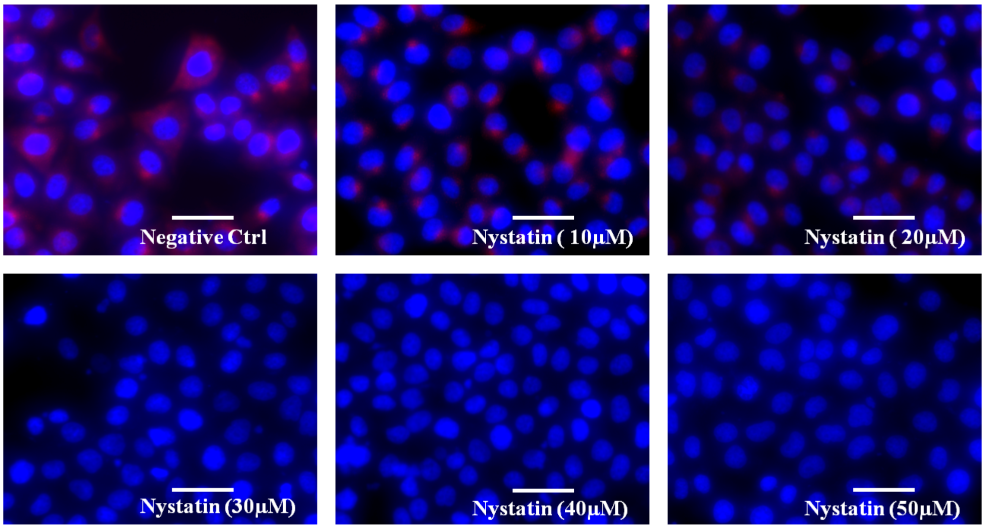

S1. CTB uptake was inhibited by nystatin. HeLa cells were treated with nystatin for 1 h and then incubated with 20 μg/ml Alexa Fluor 555-labeled CTB for 30 min at 37 °C, noninternalized CTB-AF555 were removed by washing with 0.1 M glycine, 0.1 M NaCl at pH 3.0 and then washing with cold PBS for three times. Cells were fixed and DAPI stained. Scale bar = 50 μm.
